# Clinical Profile and Adverse Effects of Dolutegravir Treatment in HIV-Positive Patients: A Prospective Observational Study

**DOI:** 10.7759/cureus.62522

**Published:** 2024-06-17

**Authors:** Kavita Joshi, Shital Shinde, Shifa Karatela, Alhad Mulkalwar

**Affiliations:** 1 Department of Medicine, Seth Gordhandas Sunderdas Medical College and King Edward Memorial Hospital, Mumbai, IND; 2 Department of Medicine, Government Medical College and Hospital Miraj, Sangli, IND; 3 Department of Medicine, Medical College Baroda and Sir Sayajirao General Hospital, Vadodara, IND; 4 Department of Pharmacology, Dr. D.Y. Patil Medical College, Hospital and Research Centre, Pune, IND

**Keywords:** art adverse effects, art treatment adherence, antiretroviral therapy, dolutegravir, person living with hiv/aids (plha)

## Abstract

Background

The emergence of dolutegravir (DTG) within antiretroviral therapy (ART) has drastically improved the management of HIV/AIDS, marking a shift toward a chronic manageable condition. Nevertheless, concerns persist regarding the real-world tolerability and adverse effects (AEs) of DTG.

Objective

This study aims to explore the clinical characteristics, adverse reactions, and adherence to treatment with DTG among HIV-positive individuals.

Methods

Through a prospective approach, we examined HIV-positive patients undergoing DTG-based ART regimens. Key parameters, including socio-demographic data, treatment adherence, and clusters of differentiation 4 (CD4) count, were evaluated. Enrolled patients were followed up for six months for the development of comorbidities and AEs.

Results

Initial observations indicate successful viral suppression and enhanced CD4 counts with DTG-based regimens, t(318)=2.0664, p=0.0392. However, a subset of participants experienced AEs such as neuropsychiatric symptoms (headaches and mood fluctuations), unintended weight gain, and other comorbidities linked to prolonged ART usage.

Conclusion

While DTG-based therapies offer substantial advantages in HIV/AIDS management, such as rapid viral suppression and reduced toxicity, ongoing vigilance for adverse effects, particularly neuropsychiatric symptoms and metabolic disturbances, is imperative for optimizing patient care. Further research is necessary to fully elucidate the safety profile of DTG in real-world scenarios and mitigate potential adverse reactions.

## Introduction

The first HIV case was identified in June 1981 in Los Angeles, USA, marking the start of significant advancements in HIV/AIDS prevention, diagnosis, care, and treatment globally. These include the development of newer, less toxic drug molecules, reduced therapy costs, and innovative service delivery methods, transforming the disease from a fatal condition in the 1980s to a manageable chronic illness. Over the last two decades, wider access to affordable, more effective, and less toxic antiretroviral drugs (ARVs) has led to a rapid decline in HIV-related mortality and morbidity. Antiretroviral therapy (ART), involving a combination of ARV drugs from different classes, suppresses viral replication, restores immune function, and improves quality of life [1].

While ART effectively reduces viral load and restores immune function, it cannot cure HIV. The primary goals are sustained viral load reduction and immune function restoration to reduce transmissibility and new infections [2]. Dolutegravir (DTG) is recommended by National AIDS Control Organization (NACO), as the preferred treatment for HIV-positive individuals due to its efficacy against both HIV types 1 and 2. DTG inhibits HIV replication by blocking integrase activity. It is preferred in both first-line and second-line treatment regimens [1].

Effective suppression of HIV replication plays a crucial role in extending the lifespan and enhancing the quality of life for individuals living with HIV. Significant advancements have been made in ART over recent decades. Presently, numerous countries are either incorporating or considering the inclusion of DTG-containing regimens in their national protocols, as the preferred first-line option [3].

DTG is prioritized as the first-line treatment due to its advantages, including rapid viral suppression, fewer side effects and toxicities, minimal drug interactions, effectiveness against HIV-2, and a high genetic barrier. According to the NACO guidelines of 2020 in India, there is a shift toward DTG-based regimens for both virally suppressed and non-suppressed people living with HIV (PLHIV) [1].

Given the evolving drug profiles of HIV patients, changes in clinical course, and drug side effect profiles, the adoption of new ART regimens for HIV has the potential to enhance clinical outcomes for patients. ART effectively reduces plasma viral load and extends AIDS-free survival. However, ART has two primary drawbacks: toxicity and low compliance. Persistent side effects often lead to poor adherence, which can result in suboptimal therapy or treatment discontinuation, ultimately leading to treatment failure. Recent systematic reviews and meta-analyses conducted by the World Health Organization (WHO) have indicated that DTG-based regimens are better tolerated and have a lower risk of treatment discontinuation due to adverse effects (AEs) compared to efavirenz (EFV) [4].

Despite the favorable safety profile observed in clinical trials, recent data from observational studies have raised concerns about the tolerability of DTG, with reports of treatment discontinuations due to toxicity [5]. While these findings are not universally confirmed, further studies are necessary to accurately assess the safety and tolerability profile of DTG-based regimens in real-world settings. Post-marketing surveillance has identified DTG-related adverse drug events (ADEs) in PLHIV, including neuropsychiatric AEs such as headaches, dizziness, sleep disturbances, mood changes, suicidal thoughts, cognitive issues, and unintentional weight gain [6,7]. A study by Sekar et al. reported a maximum number of ADEs with DTG compared to a non-DTG regimen [8].

As patients with HIV are now living longer, they face an increasing prevalence of age-related comorbidities, including renal, cardiovascular, and liver diseases; cognitive decline; metabolic disorders like diabetes and dyslipidemia; and osteoporosis. Adverse events associated with long-term ART use may contribute to the development of these comorbidities [5].

Therefore, it is crucial to investigate the clinical characteristics of HIV-positive patients undergoing DTG treatment. This study aims to examine the clinical profile of HIV-positive individuals receiving DTG and assess the AEs associated with DTG usage.

## Materials and methods

A prospective observational study was conducted at the ART center of King Edward Memorial Hospital, a tertiary care hospital in Western India, with approval from the institutional ethics committee (approval number EC/113/2021). The study spanned 1.5 years and included 319 participants meeting specific inclusion and exclusion criteria. The sample size was calculated using Cochran’s formula with the HIV prevalence of 36% in Maharashtra [9]. All the patients registered at the ART center receiving the DTG regimen, aged >18 years, and willing to be enrolled by signing the informed consent document were included as study participants. Patients receiving TLD (Tenofovir + Lamivudine + Dolutegravir) as a post-exposure prophylaxis medication and pregnant females were excluded. Our primary objective was to assess ADEs among DTG users and our secondary objective was treatment adherence and immunologic improvement with DTG.

After approval of IEC, the study commenced with an enrolment of HIV-positive patients receiving TLD regimens. A total of 319 patients fulfilling inclusion criteria were enrolled in the study. Enrolled patients were informed about the nature of the study and the non-disclosure of their sensitive information. All study participants receiving DTG were asked for their previous drug history, whether they were ART-naive or ART-experienced. Those with prior ART experience were specifically asked about their previous regimens. The change in regimen was carried out by the treating physician in accordance with updated guidelines and was not performed for the purpose of the study [10]. A comprehensive assessment, with detailed history and examinations, was documented on a predesigned proforma. Sociodemographic parameters such as age, gender, and socioeconomic status; physical examination findings of weight, height, and BMI (body mass index) (cut off according to Asia Pacific classification); and hematologic examinations including complete blood count (CBC), liver function tests (LFT), renal function test (RFT), blood glucose tests: fasting blood sugar (FBS) and postprandial blood sugar (PPBS), fasting lipid profile, and clusters of differentiation (CD4) count were noted [11]. The socioeconomic status of patients was graded by the Modified Kuppuswamy scale, a 29-point-based system dividing patients into five classes [12]. Enrolled patients were followed up for six months for the development of comorbidities and AEs.

The collected data was compiled using Microsoft Excel 2010 (Microsoft Corporation, Redmond, Washington) and analyzed with SPSS V 23 (IBM Corporation, Armonk, New York). A descriptive analysis was performed. Numerical data like hemodynamic parameters (pulse rate and systolic and diastolic blood pressure), CBC, LFT, RFTs, blood sugar levels, lipid profile, and CD4 count were expressed as means and standard deviation (SD). A comparison between before DTG and after DTG groups was performed using Student’s paired t-test for all the quantitative parameters mentioned above. Categorical data like age group, gender, socioeconomic classes, BMI, comorbidities, ADEs, and previous exposure to ART were described using numbers and percentages. The association of age group, gender, BMI, comorbidities individually with ADEs, weight gain, hyperglycemia, hyperlipidemia, and elevated liver enzymes, treatment adherence were analyzed using the Chi-Square test as appropriate. A two-sided p-value of <0.05 was considered significant.

## Results

The majority of the study subjects belonged to the age group of 41-50 years (40.8%). The male-to-female ratio in the study was 1.02:1. The majority belonged to the lower middle socioeconomic class (121 (37.9%)). Table 1 shows the distribution of study subjects according to age, gender, and socioeconomic class.

**Table 1:** Distribution of age, gender, and socioeconomic class of patients receiving DTG * The modified Kuppuswamy class is a criterion to grade socioeconomic status based on a 29-point system, allocating 12 points for family income, 10 points for occupation, and seven points for education [12]. DTG: dolutegravir

Age group (years)	No. of patients
18-30	29 (9.1%)
31-40	44 (13.8%)
41-50	130 (40.8%)
51-60	101 (31.7%)
61-70	12 (3.8%)
71-80	3 (0.9%)
Gender	No. of patients
Male	161 (50.5%)
Female	158 (49.5%)
Modified Kuppuswamy class*	No. of patients
Upper (26-29)	1 (0.3%)
Upper middle (16-25)	55 (17.2%)
Lower middle (11-15)	121 (37.9%)
Upper lower (5-10)	88 (27.6%)
Lower (<5)	54 (16.9%)
Total	319 (100%)

Table 2 shows the distribution of comorbidities among study subjects. Tuberculosis (TB), n=99 (31.0%), was the most common comorbidity found among HIV-positive study participants.

**Table 2:** Distribution of comorbidities among patients receiving DTG DTG: dolutegravir; DM: diabetes mellitus; TB: tuberculosis; IHD: ischemic heart disease; HTN: hypertension

Comorbidities	No. of patients
TB	99 (31.0%)
HTN	77 (24.1%)
DM	42 (13.2%)
Bronchial asthma	6 (1.9%)
Thyroid disorder	4 (1.3%)
IHD	4 (1.3%)

Although more patients turned overweight or obese than underweight after starting DTG, no significant association could be elicited between BMI and DTG use as shown in Table 3.

**Table 3:** Distribution of BMI in study subjects before and after receiving DTG Classification criteria and its range: BMI according to Asia-Pacific classification, underweight (<18.5 kg/m^2^), normal (18.5 kg/m^2^-22.9 kg/m^2^), overweight (23.0 kg/m^2^-24.9 kg/m^2^), and obese (≥25 kg/m^2^). BMI: body mass index; DTG: dolutegravir; DOF: degrees of freedom

BMI category (kg/m^2^)	Before DTG	After DTG	X^2^ (DOF, n)	P-value
Underweight	34 (10.7%)	26 (8.2%)	1.177 (1, 638)	0.277
Normal	139 (43.6%)	136 (42.6%)	0.057 (1, 638)	0.810
Overweight	57 (17.9%)	61 (19.1%)	0.166 (1, 638)	0.683
Obese	89 (27.9%)	96 (30.1%)	0.373 (1, 638)	0.541

Previous ART exposure was assessed among study subjects. The majority n=295 (92.5%) were previously exposed to ART, and n=24 (7.5%) were ART-naive and were started on a DTG-based ART regimen. However, in all the patients, the previous regimen was changed by the treating physician, in view of the change in guidelines, and not specifically for the study (Table 4) [10].

**Table 4:** ART regimen change among study participants ART: antiretroviral therapy

Previous regimen without dolutegravir	Current regimen containing dolutegravir	Number of patients
Tenofovir + Lamivudine + Efavirenz (TLE)	Tenofovir + Lamivudine + Dolutegravir (TLD)	179
Zidovudine + Lamivudine + Nevirapine (ZLN)	Tenofovir + Lamivudine + Dolutegravir (TLD)	79
Tenofovir + Lamivudine + Atazanavir/Ritonavir (TL ATV/R)	Tenofovir + Lamivudine + Dolutegravir (TLD)	18
Tenofovir + Lamivudine + Lopinavir/Ritonavir (TL LPV/R)	Tenofovir + Lamivudine + Dolutegravir (TLD)	10
Zidovudine + Lamivudine + Efavirenz (ZLE)	Tenofovir + Lamivudine + Dolutegravir (TLD)	6
Abacavir + Lamivudine + Efavirenz (ALE)	Abacavir + Lamivudine + Dolutegravir (ALD)	2
Tenofovir + Lamivudine + Efavirenz (TLE)	Abacavir + Lamivudine + Dolutegravir(ALD)	1

**Figure 1:**
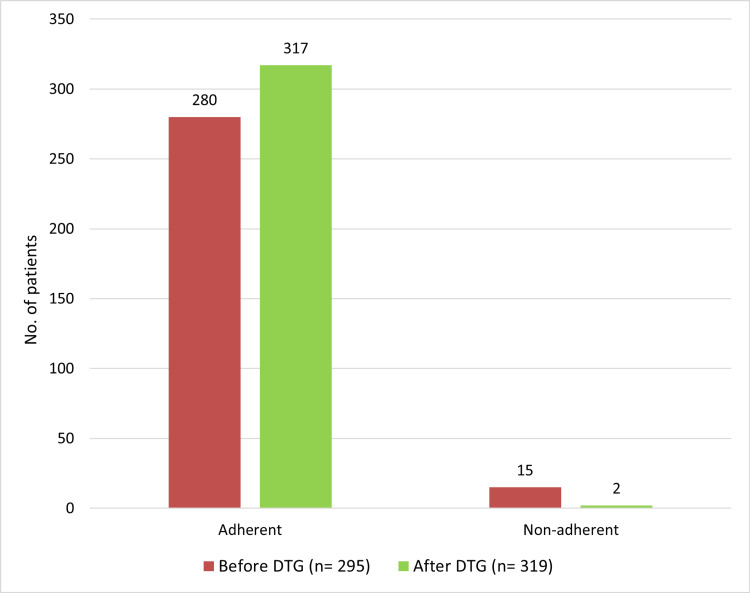
Adherence among HIV-positive patients before and after receiving DTG DTG: dolutegravir

A strong and statistically significant association was found in treatment adherence before and after DTG initiation as shown in Figure 1. *X*^2^(1, N=614)=11.3135, p <0.01 that is treatment adherence increased after switching to DTG.

**Figure 2:**
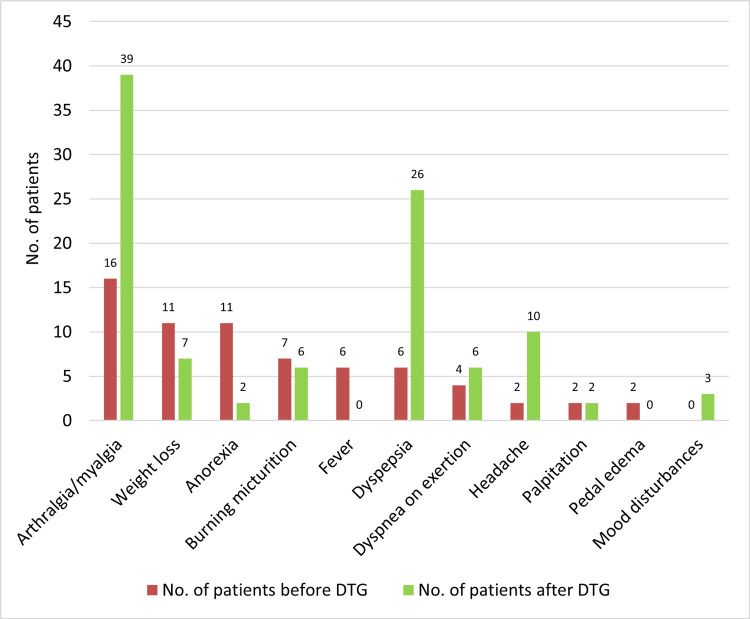
Clinical features of the study subjects before and after receiving DTG DTG: dolutegravir

Figure 2 shows the clinical features of HIV-positive patients before receiving DTG and six months after DTG. There were new-onset mood disturbances in three (0.94%) study subjects receiving DTG.

Table 5 shows the distribution and assessment of vital signs, CBC, RFT, liver enzymes, blood sugar levels, lipid profile, and CD4 T-cell count in HIV-positive patients before and after receiving DTG. We observed that bilirubin levels in all study subjects were in the non-icteric range (NICT). There was a statistically significant increase in the mean values of WBC, liver enzymes, lipid profile, and blood sugar levels along with an increase in mean CD4 count in patients after initiating DTG, p-value<0.05.

**Table 5:** Distribution and assessment of vitals and blood parameters in patients before and after receiving DTG Statistically significant results are in bold numbers. DTG: dolutegravir; SD: standard deviation; SBP: systolic blood pressure; DBP: diastolic blood pressure; Hb: hemoglobin; WBC: white blood cell; BUN: blood urea nitrogen; SGOT: serum glutamic oxaloacetic transaminase; SGPT: serum glutamic pyruvic transaminase; FBS: fasting blood sugar; PPBS: postprandial blood sugar; Chol: cholesterol; TG: triglycerides; CD4: clusters of differentiation 4

Parameter	Before DTG: mean (SD)	After DTG: mean (SD)	t-test	P-value
Pulse rate (beats/min)	82.66 (9.75)	82.10 (8.83)	0.7604	0.4473
SBP (mm Hg)	116.12 (10.92)	116.61 (13.61)	0.5015	0.6162
DBP (mm Hg)	75.14 (10.82)	76.02 (11 .70)	0.9863	0.3244
Hb (g/dL)	12.00 (2.03)	12.13 (1.98)	0.8188	0.4132
WBC (cells/μL)	6970 (2025)	7313 (2310)	1.9942	0.0466
Platelet (lac/μL)	2.56 (0.74)	2.57 (0.75)	0.1695	0.8654
BUN (mg/dL)	10.32 (2.92)	10.22 (2.97)	0.4288	0.6682
Creatinine (mg/dL)	1.22 (0.51)	1.34 (1.96)	1.0583	0.2903
SGOT (U/L)	29.76 (8.14)	38.90 (32.82)	4.8277	<0.01
SGPT (U/L)	27.90 (13.10)	39.66 (38.68)	5.1432	<0.01
FBS (mg/dL)	91.28 (19.85)	95.62 (26.79)	2.3248	0.020
PPBS (mg/dL)	127.45 (40.53)	138.18 (51.40)	2.9278	<0.01
Chol (mg/dL)	165.76 (30.4)	175.8 (45.32)	3.286	<0.01
TG (mg/dL)	96.97 (32.30)	121.71 (76.17)	5.3408	<0.01
CD4 count (cells/mm^3^)	544.77 (216.49)	581.04 (226.74)	2.0664	0.0392

**Figure 3:**
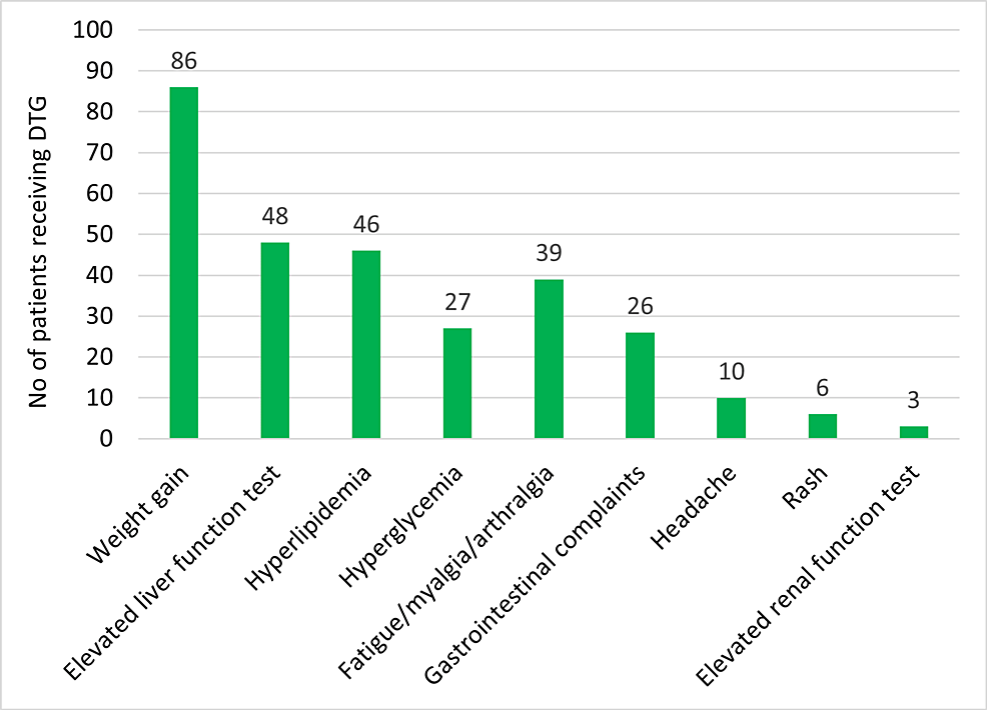
Frequency distribution of ADEs in patients receiving DTG DTG: dolutegravir; ADE: adverse drug event

Figure 3 shows the frequency distribution of ADEs in patients receiving DTG. Weight gain was the most common drug-related adverse event among study subjects (n=86), followed by elevated liver function (n=48), hyperlipidemia (n=46), and hyperglycemia (n=27), respectively.

Table 6 shows the distribution and association between the number of ADEs and age, gender, BMI, ART exposure, diabetes mellitus (DM), and hypertension (HTN). No significant association of age, gender, BMI, or ART exposure existed with the number of adverse events. A statistically significant association of the number of ADEs existed with diabetes and HTN (p-value <0.05) that is diabetes and HTN influenced the development of ADEs among DTG users.

**Table 6:** Distribution and association of ADEs with demographic and clinical parameters ADE: adverse drug event; ART: antiretroviral therapy; DM: diabetes mellitus; BMI: body mass index; HTN: hypertension

	ADEs		
	No ADE	Any 1 ADE	≥2 ADE
Age			
18-30 years (n=29)	19 (65.5%)	8 (27.6%)	2 (6.9%)
31-40 years (n=44)	19 (43.2%)	16 (36.4%)	9 (20.5%)
41-50 years (n=130)	52 (40.0%)	52 (40.0%)	26 (20.0%)
51-60 years (n=101)	36 (35.6%)	42 (41.6%)	23 (22.8%)
61-70 years (n=12)	7 (58.3%)	2 (16.7%)	3 (25.0%)
71-80 years (n=3)	2 (66.7%)	0 (0.0%)	1 (33.3%)
X^2^(10, N=319)=13.30, p=0.207			
Gender			
Male	72 (53.3%)	59 (49.2%)	30 (46.9%)
Female	63 (46.7%)	61 (50.8%)	34 (53.1%)
X^2^(2, N=319)=0.8552, p=0.65			
BMI categories			
Underweight (n=26)	12 (8.9%)	13 (10.8%)	1 (1.6%)
Normal (n=136)	59 (43.7%)	51 (42.5%)	26 (40.6%)
Overweight (n=61)	28 (20.7%)	20 (16.7%)	13 (20.2%)
Obese (n=96)	36 (26.7%)	36 (30.0%)	24 (37.6%)
X^2^ (6, N=319)=6.96, p=0.324			
Previous exposure			
ART exposed (n=295)	124 (91.9%)	111 (92.5%)	60 (93.75%)
ART naïve (n=24)	11 (8.1%)	9 (7.5%)	4 (6.25%)
X^2^(2, N=319)=1.68, p=0.43			
DM			
Diabetic	8 (5.9%)	15 (12.5%)	19 (29.7%)
Non-diabetic	127 (94.1%)	105 (87.5%)	45 (70.3%)
X^2^(2, N=319)=21.517, p<0.01			
HTN			
Hypertensive	21 (15.6%)	36 (30.0%)	20 (31.3%)
Non-hypertensive	114 (84.4%)	84 (70.0%)	44 (68.8%)
X^2^(2, N=319)=9.450, p<0.01			

Table 7 shows the distribution and association of hyperlipidemia with age and gender. A statistically significant association of hyperlipidemia existed with age and gender. In this study, we assessed the response to treatment among study subjects having hyperlipidemia, p-value <0.05. Among 46 study subjects having hyperlipidemia, 27 were started on treatment (statins), and 20 were not receiving any treatment for hyperlipidemia. Among, 27 who were started on treatment (statins) for hyperlipidemia 22 subjects (80.76%) showed improvement in lipid profile; however, five subjects (19.24%) failed to show improvement in lipid profile.

**Table 7:** Distribution and association of hyperlipidemia with age and gender

	Hyperlipidemia	
	Yes	No
Age group		
18-30 years (n=29)	0 (0.0%)	29 (100.0%)
31-40 years (n=44)	1 (2.3%)	43 (97.7%)
41-50 years (n=130)	25 (19.2%)	105 (80.8%)
51-60 years (n=101)	18 (17.8%)	83 (82.2%)
61-70 years (n=12)	2 (16.7%)	10 (83.3%)
71-80 years (n=3)	0 (0.0%)	3 (100.0%)
Total (n=319)	46 (14.4%)	273 (85.6%)
X^2^(5, N=319)=14.087, p=0.015		
Gender		
Males (n=161)	17 (10.6%)	144 (89.4%)
Female (n=158)	29 (18.4%)	129 (81.6%)
X^2^(1, N=319)=3.927, p=0.047		

Out of 319 participants, 27 study subjects receiving DTG had hyperglycemia. Among 27 subjects, 14 (51.85%) were known cases of diabetes in whom there was a derangement in blood sugar levels, and 13 (48.15%) were having new-onset hyperglycemia. Among 13 patients who developed new-onset hyperglycemia, 10 were males, and among 14 patients who had known cases of DM who had worsening of sugar profile, nine were males. Table 8 shows age, gender, and BMI distribution and their association with hyperglycemia. Hyperglycemia was positively associated with age and gender but not with BMI.

**Table 8:** Distribution and association of hyperglycemia with age, gender, and BMI BMI: body mass index

	Hyperglycemia	
	Yes	No
Age group		
18-30 years (n=29)	2 (6.9%)	27 (93.1%)
31-40 years (n=44)	3 (6.8%)	41 (93.2%)
41-50 years (n=130)	8 (6.2%)	122 (93.8%)
51-60 years (n=101)	8 (7.9%)	93 (92.1%)
61-70 years (n=12)	5 (41.7%)	7 (58.3%)
71-80 years (n=3)	1 (33.3%)	2 (66.7%)
Total (n=319)	27 (8.4%)	292 (91.5%)
X^2^(5, N=319)=20.65, p<0.01		
Gender		
Male (n=161)	19 (11.8%)	142 (88.2%)
Female (n=158)	8 (5.1%)	150 (94.9%)
X^2^(1, N=319)=4.673, p=0.043		
BMI		
Underweight (n=26)	2 (7.7%)	24 (92.3%)
Normal (n=136)	10 (7.4%)	126 (92.6%)
Overweight (n=61)	3 (4.9%)	58 (95.1%)
Obese (n = 96)	12 (12.5%)	84 (87.5%)
X^2^(3, N=319)=3.2451, p=0.3553		

We assessed the treatment history among patients having hyperglycemia. None of the patients were started on insulin. Table 9 shows the distribution of treatment initiation in patients with hyperglycemia. In known cases of DM with deteriorating sugar levels, additional oral hypoglycemic agents (OHA) were introduced. Conversely, in newly diagnosed cases of DM, initiation of fresh OHA was done. However, we were not able to assess the response of OHAs in these patients because of reasons whiz limited study duration, non-availability of FBS, and PPBS reports after starting treatment, and noncompliance to treatment.

**Table 9:** Age-wise diabetes treatment distribution in patients receiving DTG DM: diabetes mellitus; DTG:dolutegravir

Age group	New-onset DM (treatment received: yes/no)	Known cases of DM (treatment received: yes/no)
18-30 years (n=2)	2 (1-yes, 1-no)	0 (0-yes, 0-no)
31-40 years (n=3)	0 (0-yes, 0-no)	3 (3-yes, 0-no)
41-50 years (n=8)	2 (1-yes, 1-no)	6 (6-yes, 0-no)
51-60 years (n=8)	6 (1-yes, 5-no)	2 (2-yes, 0-no)
61-70 years (n=5)	3 (3-yes, 0-no)	2 (2-yes, 0-no)
71-80 years (n=1)	0 (0-yes, 0-no)	1 (1-yes, 0-no)
Total (n=27)	13 (6-yes, 7-no)	14 (14-yes, 0-no)

Table 10 shows the distribution and association of elevated liver enzymes with age, gender, and BMI. Among 48 study subjects having elevated liver enzymes, 37 study subjects (77.02%) had self-resolution of elevated liver enzymes, and 11 study subjects (22.98%) had persistent liver enzyme elevation. In this study, we observed that three among 319 subjects had HbsAg-positive status. All three were males. Among three HbsAg-positive patients, two had normal liver enzymes and one had elevated liver enzymes; however, it was self-resolving and not needed to change DTG. Elevation of liver enzymes after initiating DTG was not associated with age, gender, or BMI.

**Table 10:** Distribution and association of elevated liver enzymes with age, gender, and BMI BMI: body mass index

	Elevated liver enzymes	
	Yes	No
Age group		
18-30 years (n=29)	7 (24.1%)	22 (75.9%)
31-40 years (n=44)	10 (22.7%)	34 (77.3%)
41-50 years (n=130)	15 (11.5%)	115 (88.5%)
51-60 years (n=101)	16 (15.8%)	85 (84.2%)
61-70 years (n=12)	0 (0.0%)	12 (100.0%)
71-80 years (n=3)	0 (0.0%)	3 (100.0%)
Total (n=319)	48 (15.04%)	271 (85.74%)
X^2^(5, N=319)=7.86, p=0.18		
Gender		
Males (n=161)	25 (15.5%)	136 (84.5%)
Females (n=158)	23 (14.6%)	135 (85.4%)
X^2^(1, N=319)=0.059, p=0.876		
BMI		
Underweight (n=26)	4 (15.4%)	22 (84.6%)
Normal (n=136)	23 (16.9%)	113 (83.1%)
Overweight (n=61)	6 (9.8%)	55 (90.2%)
Obese (n=96)	15 (15.6%)	81 (84.4%)
X^2^(3, N=319)=1.693, p=0.638		

Table 11 shows the distribution and association of weight gain with age and gender among study subjects. No significant association existed.

**Table 11:** Distribution and association of weight gain with age and gender

	Weight gain	
	Yes	No
Age group		
18-30 years (n=29)	2 (6.9%)	27 (93.1%)
31-40 years (n=44)	14 (31.8%)	30 (68.2%)
41-50 years (n=130)	40 (30.8%)	90 (69.2%)
51-60 years (n=101)	26 (25.7%)	75 (74.3%)
61-70 years (n=12)	3 (25.0%)	9 (75.0%)
71-80 years (n=3)	1 (33.3%)	2 (66.7%)
Total (n=319)	86 (26.9%)	233 (73.1%)
X^2^(5, N=319)=7.57, p=0.105		
Gender		
Males (n=161)	41 (25.5%)	120 (74.5%)
Female (n=158)	45 (28.5%)	113 (71.5%)
X^2^(1, N=319)=0.368, p=0.315		

There was a significant association between diabetes and weight gain (p-value=0.041). However, we could not find any significant association between weight gain and other comorbidities (Table 12).

**Table 12:** Association between weight gain and comorbidities Statistically significant results are in bold numbers. HTN: hypertension; IHD: ischemic heart disease; DM: diabetes mellitus; TB: tuberculosis

Comorbidities	Weight gain		P-value
	Yes	No	
DM (n=42)	17 (40.5%)	25 (59.5%)	0.041
HTN (n=77)	25 (32.5%)	52 (67.5%)	0.239
Thyroid disorder (n=4)	0 (0.0%)	4 (100.0%)	0.577
TB (n=99)	26 (26.3%)	73 (73.7%)	0.892
Bronchial asthma (n=6)	2 (33.3%)	4 (66.7%)	0.662
IHD (n=4)	0 (0.0%)	4 (100.0%)	0.577

## Discussion

According to India's HIV Estimation Report 2020, the country's adult HIV prevalence (15-49 years) was 0.22% in 2020. From a projected peak level of 0.54% in 2000-2001, 0.32% in 2010, to 0.21% in 2020, the national adult prevalence continued to decrease [13]. In our study, a lesser number of study subjects belonged to the age group 18 to 30 and 31 to 40 age group, compared to the 41 to 50 age group and the 51 to 60 age group. This correlates with the decreasing prevalence of the disease [1]. The goal of NACP-current V's phase (2021-2026) is to ensure that PLHIV live longer, productive lives. This will be accomplished via increasing introduction to HIV treatment and ART compliance [1].

Initiating ART promptly after HIV diagnosis is crucial to suppress the virus effectively, minimize immunologic damage, and reduce the risk of transmission. Early treatment initiation improves clinic attendance and hinders ongoing HIV replication, which can produce billions of viral particles. The availability of more effective and less toxic ART has led to a significant decline in HIV-related mortality and morbidity over the past decades [14].

ART involves using a combination of at least three ARVs from different classes to inhibit HIV replication and decrease viremia to undetectable levels. By suppressing viral replication, ART restores the immune system, as evidenced by an increase in CD4 count. Higher CD4 counts lead to slower disease progression, fewer opportunistic infections, improved quality of life, and increased lifespan. Successful ART has transformed the perception of HIV infection from a "virtual death sentence" to a "chronic manageable illness" [15-17].

Monitoring the laboratory values of plasma HIV RNA (viral load) and CD4+T cell count in addition to the patient's clinical condition should serve as a guide for decisions on the commencement or modification of ART. Clinical professionals can use the findings of these laboratory tests to determine a patient's virologic and immunologic state and the likelihood that their condition will proceed to AIDS.

While nucleoside reverse transcriptase inhibitors (NRTIs) have long been considered fundamental in ART, concerns have emerged regarding their toxicity, particularly with older agents like stavudine and zidovudine and newer ones like tenofovir and abacavir. NRTIs, including older agents like stavudine and zidovudine, have been associated with serious mitochondrial toxicity and other AEs such as anemia and peripheral neuropathy. Newer agents like tenofovir and abacavir have been linked to nephrotoxicity, bone mineral density loss, and potentially cardiovascular events. These concerns have prompted the exploration of NRTI-sparing regimens. The recycling of NRTIs in patients who have failed previous regimens raises concerns about the development of resistance and decreased drug potency. This is particularly problematic in resource-limited settings where options for alternative medications are limited. The emergence of newer agents from different classes, such as integrase inhibitors, non-nucleoside reverse transcriptase inhibitors (NNRTIs), CCR5 inhibitors, and protease inhibitors, offers alternatives to NRTIs. These agents provide opportunities for NRTI-sparing regimens that maintain efficacy while potentially reducing toxicity. Switching from a damaged NRTI backbone to alternative agents requires careful consideration to avoid treatment failure. Utilizing regimens with two to three fully active drugs from different classes can help maintain treatment efficacy while minimizing toxicity. Overall, the evolving understanding of NRTI toxicity and the availability of newer agents are driving the exploration of NRTI-sparing regimens in HIV treatment, aiming to balance efficacy and safety considerations [18-20].

ART is significantly improved by the inclusion of integrase inhibitors. Integrase strand transfer inhibitors (INSTIs) are now viewed as an alternative "third agent" class of drug that works by inhibiting the HIV-1 integrase enzyme, which has lately emerged as a prominent alternative target to prevent viral replication. It includes raltegravir, elvitegravir, and DTG.

DTG is the first next-generation INSTI to exhibit several unusual and intriguing properties. DTG is the newest HIV weapon in the developed world thanks to its special qualities, including a once-daily dose for ART-naive patients, a long intracellular half-life, lack of cross-resistance to first-generation INSTI, high genetic barrier to resistance, and excellent safety profile and unboosted daily dose. For the majority of ART and other routinely co-administered medications, DTG has shown a small number of clinically relevant drug-drug interactions without dose change. At normal quantities, DTG neither induces nor inhibits CYP450 enzymes or UGT1A1. DTG can be dosed without taking into account food because these changes in exposure are not anticipated to influence safety or effectiveness [21].

DTG has regularly been found in studies to result in lower rates of discontinuation than EFV. Tenofovir, lamivudine, and EFV users have significantly higher rates of quitting all ART than users of tenofovir, lamivudine, and DTG, while their benefits are probably greater as well. Long-term medication side effects, including weight gain, and their impact on ART use patterns should be closely evaluated. The majority of participants preferred DTG-based ART's once-daily morning dose and tiny tablet size; however, many admitted it was difficult for them to get used to a new dosing schedule. Participants relished changing to a medication that was more advantageous based on the MoH's recommendations. This study expands on earlier studies that demonstrated the superiority of DTG over existing treatment regimens and its more manageable side-effect profile. The potential direct health advantages of DTG over first-line EFV-based regimens for patients, including increased pre-treatment resistance to NNRTIs, fewer side effects, quick viral suppression, and low pill burden, were the driving forces behind drug switching. Pill burden, such as the quantity of tablets taken daily or the size of the tablets, is linked to poor adherence. Because DTG has fewer pills to take, individuals might stick to their treatment plans better [22,23].

Additionally, generic fixed-dose combinations of DTG-based regimens are now readily available, and there is more assurance that they will be used in crucial sub-populations, increasing confidence in the selection of this regimen as the recommended ARV treatment for a public health approach [24].

Due to the associated wasting, there is a general understanding that PLHIV are mostly underweight. Hence HIV infection is referred to as the "slim disease." However, with the increased availability of ART, there is immunological improvement in PLHIIV subjects. It has led to a decrease in opportunistic infections and an improvement in the clinical profile of PLHIV, resulting in weight gain in PLHIV. Some ART drugs like DTG affect metabolic parameters in PLHIV leading to weight gain. All these factors contribute to an increase in BMI in PLHIV over some time. This has led to most subjects belonging to the normal or overweight BMI category compared to a previous era, where PLHIV subjects were underweight as observed in our study; 42.6% of study subjects had normal BMI, 8.2% of study subjects were underweight, 19.1% of study subjects were overweight, and 30.1% of study subjects were obese [7].

Low socioeconomic class and its implications include reduced educational success, poverty, and bad health. Globally there are growing inequalities in the distribution of resources, health, and standard of living. The majority of the subjects in our study belonged to lower socioeconomic classes.

According to Riley ED et al., a lack of socioeconomic resources is linked to the practice of riskier health behaviors, which can lead to the contraction of HIV. Additionally, Socioeconomic status plays a significant role in influencing an individual's quality of life after contracting the virus. Limited economic opportunities and lack of education have been associated with risky sexual practices, such as exchanging sex for money. HIV risk for men and women may vary depending on socioeconomic characteristics. Male HIV risk has been linked to income inequality, while female HIV risk has been linked to poverty, health, and housing conditions [25].

The majority of the subjects in our study had past/current TB (31.0%), 24.1% subjects were hypertensive, 13.2% subjects had DM, 1.9% study subjects had bronchial asthma, 1.3% subjects had thyroid disorder, and 1.3% subjects had ischemic heart disease (IHD).

Poor immunity, low socioeconomic status, and poor nutrition could be contributing factors to the higher rate of TB in PLHIV. According to Majigo M et al., the incidence and prevalence of TB in PLHIV patient studies in Tanzania, early initiation of ART, and the provision of TB preventive therapy for those PLHIV without active TB have led to a decrease in the incidence of TB in PLHIV [26].

With the introduction of ART, there is an increase in the lifespan of PLHIV, and the burden of non-HIV-related comorbidities is increasing. Christensen S et al. study results showed that PLHIV patients had higher rates of comorbid conditions than people with similar characteristics from the general population. PLHIV had an increased risk of cardiovascular disease (CVD), acute and chronic renal illness, and osteoporosis-related bone fractures, with earlier onset of these disorders. The latest ART treatment choices lengthen PLHIV's life expectancy; however, HIV patients appear to be more susceptible to age-related comorbidities when compared to a cohort of patients with similar features who were not affected by HIV [27].

In this study, we assessed the AEs of DTG among the study subjects. The majority of the study subjects showed no AEs (42.3%), 37.6% of study subjects had one AE, and 20.1% of study subjects had more than or equal to two AEs. In this study, we observed weight gain as the most common ADE, followed by elevated LFT, hyperlipidemia, hyperglycemia, myalgia, and headache, respectively.

In Hongo’s DTG ADE study in Japan, the incidence of ADE was 24.45%. In this case, raised creatinine was the most common ADE that was followed by neuropsychiatric ADE, followed by weight gain. Nausea and diarrhea were also frequently reported ADEs [28].

In this study, we assessed the association between co-morbidities among study subjects receiving DTG and ADEs. We observed that there is a significant association between diabetes, HTN, and ADEs. However, we could not find any research regarding comorbidities in PLHIV and ADE. One of the possible causes of association between DM and ADEs and also between HTN and ADEs would be that in our study most of the ADEs were related to metabolic parameters; DM and HTN could cause an additive effect on metabolic parameter derangement.

In this study, we observed neuropsychiatric adverse events in PLHIV receiving DTG. We observed that 3.1% of study subjects had headaches, 0.84% of study subjects had mood disturbances, and 26.95% of study subjects had weight gain. The average weight gain over six months was 4.01 kg.

Weight gain is thought to be caused by a decrease in basal metabolic rate following suppression of plasma viremia, an increase in appetite brought on by a decrease in the effects of inflammatory cytokines on the hypothalamus, and a decrease in the rate of protein turnover. However, when compared with people starting elvitegravir-based regimens, it was shown that PLHA starting DTG- and raltegravir-based regimens gained much more weight. Additionally, compared to people starting NNRTI-based regimens, PLHA starting DTG-based regimens gained noticeably more weight after 18 months [29].

In a systematic review and meta-analysis on obesity and overweight among adult HIV-infected people taking ART in Ethiopia, the pooled prevalence of overweight and obesity among HIV-infected people taking ART was 17.85% and 3.9%, respectively. The finding was comparable to a large national-level cross-sectional study done in Tanzania among adult PHIV [30,22].

This poses a huge trouble among HIV patients since even without obesity, individuals living with HIV are at greater risk for non-communicable diseases like diabetes and other CVDs. Overweight and obesity were highly prevalent among HIV-infected patients. Screening for overweight and obesity and focused interventions should be integrated into HIV care. Interventions like lifestyle modification and health education to reduce the burden in this section of the population are also needed. However, in our study, we observed that only seven patients were newly added to the obese group of study subjects and there was no association between hyperglycemia and BMI in study subjects, thus weight gain could be considered a boon in our study group.

In this study, we observed hyperglycemia as an ADR. The factors contributing to hyperglycemia could include reduced glucose utilization, increased insulin resistance, and increased glucose production. According to VIKING-3, a single-armed phase III clinical trial, hyperglycemia was one of the most common laboratory abnormalities in 14% of the patients at week 48 of INSTI therapy [31].

We also observed that there is a significant association between gender among study subjects and hyperglycemia. Males had more risk of hyperglycemia. However, male sex is an independent risk factor for type 2 DM. Similarly, we observed a significant association between age group among study subjects and hyperglycemia, with increasing age associated with an increased risk of hyperglycemia. However, like male sex, age is an independent risk factor for type 2 diabetes. Thus, the association between gender and hyperglycemia among study subjects and the association between age and hyperglycemia seems spurious.

In this study, we observed dyslipidemia in PLHIV receiving DTG. We observed that 46 among 319 (14.4%) study subjects had dyslipidemia. However, 80% of study subjects having dyslipidemia responded to lipid-lowering agents.

Since our study was limited to six months, we could not find complications secondary to dyslipidemia like cerebrovascular accident and IHD. However, over a long period, dyslipidemia can result in life-threatening complications. So PLHIV started on DTG should be screened for dyslipidemia and if dyslipidemia is present, the patient should be started on lipid-lowering agents as early as possible since the majority of them had good responses to lipid-lowering agents.

In this study, we observed liver enzyme elevation in study subjects. We observed that 15% of study subjects had elevated liver enzymes. However, among PLHIV having LFT derangement, 77.02% had self-resolution of LFT to normal state without any intervention and 22.98% of study subjects continue to have LFT derangement.

In the Wohlfeiler M study in Opera Cohort, the proportion of LFT elevation was higher among RAL compared to DTG (14% and 6%, respectively) [32]. According to a study by Joshi K, there has been a staggering surge in hepatotoxicity cases linked to ARV drugs [33].

In this study, we assessed the immunological improvement in PLHIV receiving DTG. We observed that there is an increase in mean CD4 count in PLHIV after receiving DTG. The better tolerability and fewer ADEs compared to the previous ART regimen led to better adherence. This results in an improvement in CD4 count, resulting in immunological improvement in PLHIV receiving DTG. This has resulted in decreased subspeciality to infections, decreased opportunistic infections, and overall better quality of life.

Limitations of study

The present study was limited to a six-month duration, so we could not assess long-term ADEs caused by DTG. Due to limited resources, like the non-availability of FBS and PPBS reports, response to OHAs for hyperglycemia could not be assessed. The study comprises a population from western India hence a study including larger geographical demography is warranted for generalizability of results.

## Conclusions

DTG is overall a well-tolerated drug among patients living with HIV. With DTG there is improvement in adherence to ART and, hence, in immune status among PLHIV. Weight gain, hyperlipidemia, hyperglycemia, and liver enzyme derangement were the main ADEs due to DTG. A few neuropsychiatric symptoms were also reported. However, all the ADEs were manageable and none of these were life-threatening or required hospital admission. No patient required drug discontinuation due to DTG-related adverse events. Patients with HIV should be screened and treated for non-communicable diseases like DM and HTN and communicable diseases like TB.

## References

[1] (2024). National guidelines for HIV care and treatment 2021. https://naco.gov.in/sites/default/files/National_Guidelines_for_HIV_Care_and_Treatment_2021.pdf.

[2] Thate RN, Ingole DN, Solanke-Surase V, Joshi K, Bajpayi S, Acharya S, Nataraj G (2023). Role of CD4 count estimation in the era of HIV-1 viral load among PLHIV. Indian J Med Microbiol.

[3] Dorward J, Lessells R, Drain PK (2018). Dolutegravir for first-line antiretroviral therapy in low-income and middle-income countries: uncertainties and opportunities for implementation and research. Lancet HIV.

[4] (2024). Transition to new antiretroviral drugs in HIV programmes: clinical and programmatic considerations July 2017. https://iris.who.int/bitstream/handle/10665/255887/WHO-HIV-2017.23-eng.pdf?sequence=1.

[5] Chawla A, Wang C, Patton C, Murray M, Punekar Y, de Ruiter A, Steinhart C (2018). A review of long-term toxicity of antiretroviral treatment regimens and implications for an aging population. Infect Dis Ther.

[6] Mondi A, Cozzi-Lepri A, Tavelli A (2019). Effectiveness of dolutegravir-based regimens as either first-line or switch antiretroviral therapy: data from the Icona cohort. J Int AIDS Soc.

[7] Norwood J, Turner M, Bofill C (2017). Brief report: weight gain in persons with HIV switched from efavirenz-based to integrase strand transfer inhibitor-based regimens. J Acquir Immune Defic Syndr.

[8] Sekar VD, Joshi K, Bhide S (2024). Adverse drug reactions and prescription patterns of antiretroviral drugs: a longitudinal observational study from a tertiary care hospital in western India. Cureus.

[9] Kumar P, Sahu D, Rajan S (2021). District-level HIV estimates using the spectrum model in five states of India, 2017. Medicine (Baltimore).

[10] Geretti AM, Tsakiroglou M (2014). HIV: new drugs, new guidelines. Curr Opin Infect Dis.

[11] Lim JU, Lee JH, Kim JS (2017). Comparison of World Health Organization and Asia-Pacific body mass index classifications in COPD patients. Int J Chron Obstruct Pulmon Dis.

[12] Radhakrishnan M, Nagaraja SB (2023). Modified kuppuswamy socioeconomic scale 2023: stratification and updates. Int J Community Med Public Health.

[13] (2024). India HIV estimates 2021. https://naco.gov.in/sites/default/files/India%20HIV%20Estimates%202021%20_Fact%20Sheets__Final_Shared_24_08_2022_0.pdf.

[14] Saag MS (2019). HIV 101: fundamentals of antiretroviral therapy. Top Antivir Med.

[15] Omar E (2022). Antiretroviral therapy in asymptomatic early HIV. HIV Curr Res.

[16] Kemnic TR, Gulick PG (2024). HIV antiretroviral therapy. StatPearls [Internet].

[17] Dybul M, Fauci AS, Bartlett JG, Kaplan JE, Pau AK (2002). Guidelines for using antiretroviral agents among HIV-infected adults and adolescents. Ann Intern Med.

[18] Adams JL, Greener BN, Kashuba AD (2012). Pharmacology of HIV integrase inhibitors. Curr Opin HIV AIDS.

[19] Gandhi RT, Bedimo R, Hoy JF (2023). Antiretroviral drugs for treatment and prevention of HIV infection in adults: 2022 recommendations of the International Antiviral Society-USA panel. J Assoc Am Med.

[20] Achhra AC, Boyd MA (2013). Antiretroviral regimens sparing agents from the nucleoside(tide) reverse transcriptase inhibitor class: a review of the recent literature. AIDS Res Ther.

[21] Miller MM, Liedtke MD, Lockhart SM, Rathbun RC (2015). The role of dolutegravir in the management of HIV infection. Infect Drug Resist.

[22] Cahn P, Madero JS, Arribas JR (2024). Dolutegravir plus lamivudine versus dolutegravir plus tenofovir disoproxil fumarate and emtricitabine in antiretroviral-naive adults with HIV-1 infection (GEMINI-1 and GEMINI- 2): week 48 results from two multicentre, double-blind, randomised, non-inferiority, phase 3 trials. Lancet.

[23] Twimukye A, Laker M, Odongpiny EA (2021). Patient experiences of switching from Efavirenz- to Dolutegravir-based antiretroviral therapy: a qualitative study in Uganda. BMC Infect Dis.

[24] Venter WDF, Sokhela S, Simmons B (2020). Dolutegravir with emtricitabine and tenofovir alafenamide or tenofovir disoproxil fumarate versus efavirenz, emtricitabine, and tenofovir disoproxil fumarate for initial treatment of HIV-1 infection (advance): week 96 results from a randomised, phase. Lancet HIV.

[25] Riley ED, Gandhi M, Hare C, Cohen J, Hwang S (2007). Poverty, unstable housing, and HIV infection among women living in the United States. Curr HIV/AIDS Rep.

[26] Majigo M, Somi G, Joachim A (2020). Prevalence and incidence rate of tuberculosis among HIV-infected patients enrolled in HIV care, treatment, and support program in mainland Tanzania. Trop Med Health.

[27] Christensen S, Wolf E, Altevers J, Diaz-Cuervo H (2019). Comorbidities and costs in HIV patients: a retrospective claims database analysis in Germany. PLoS One.

[28] Hongo H, Nagao T, Nakamura K (2021). Safety and effectiveness analysis of dolutegravir in patients with HIV-1: interim report of post-marketing surveillance in Japan. Adv Ther.

[29] Ciccullo A, Baldin G, Borghi V (2022). Real-life impact of drug toxicity on dolutegravir tolerability: clinical practice data from a multicenter Italian cohort. Viruses.

[30] Kabthymer RH, Nega Techane S, Muche T, Ali Ewune H, Mekonnen Abate S, Feyisso Shaka M (2021). Overweight and obesity among adult HIV infected peoples receiving ART in Ethiopia: a systematic review and meta-analysis. J Prim Care Community Health.

[31] Eron JJ, Clotet B, Durant J (2013). Safety and efficacy of dolutegravir in treatment-experienced subjects with raltegravir-resistant HIV type 1 infection: 24-week results of the VIKING Study. J Infect Dis.

[32] Wohlfeiler M, Mounzer K, Brunet L (2020). Antiretroviral therapy and liver disorders in the OPERA(®) cohort. Ther Adv Drug Saf.

[33] Joshi KS, Shriwastav RR (2017). Highly active antiretroviral therapy and changing spectrum of liver diseases in HIV infected patients. Int J Res Med Sci.

